# T-Lymphocyte Deficiency Exacerbates Behavioral Deficits in the 6-OHDA Unilateral Lesion Rat Model for Parkinson’s Disease

**DOI:** 10.4172/2155-9562.1000209

**Published:** 2014-05

**Authors:** Christopher J Wheeler, Akop Seksenyan, Yosef Koronyo, Altan Rentsendorj, Danielle Sarayba, Henry Wu, Ashley Gragg, Emily Siegel, Deborah Thomas, Andres Espinosa, Kerry Thompson, Keith Black, Maya Koronyo-Hamaoui, Robert Pechnick, Dwain K Irvin

**Affiliations:** 1 Department of Neurosurgery, Cedars-Sinai Medical Center, Los Angeles, California, 90048, USA, Department of Psychiatry, Cedars-Sinai Medical Center, Los Angeles, California, 90048, USA; Occidental College, Los Angeles, CA 90041, USA; 2 Department of Biology, Occidental College, Los Angeles, CA 90041, USA; 3 Department of Basic Medical Sciences, College of Osteopathic Medicine of the Pacific Western University of Health Sciences, Pomona, CA 91766, USA

## Abstract

T-lymphocytes have been previously implicated in protecting dopaminergic neurons in the substantianigra from induced cell death. However, the role of T-cells in neurodegenerative models such as Parkinson’s disease (PD) has not been fully elucidated. To examine the role of T-lymphocytes on motor behavior in the 6-hydroxydopamine (6-OHDA) unilateral striatal partial lesion PD rat model, we assessed progression of hemi-parkinsonian lesions in the substantia nigra, induced by 6-OHDA striatal injections, in athymic rats (RNU−/−, T-lymphocyte-deficient) as compared to RNU−/+ rats (phenotypically normal). Motor skills were determined by the cylinder and D-amphetamine sulfate-induced rotational behavioral tests. Cylinder behavioral test showed no significant difference between unilaterally lesioned RNU−/− and RNU−/+ rats. However both unilaterally lesioned RNU−/− and RNU−/+ rats favored the use of the limb ipsilateral to lesion. Additionally, amphetamine-induced rotational test revealed greater rotational asymmetry in RNU−/− rats compared to RNU−/+ rats at two- and six-week post-lesion. Quantitative immunohistochemistry confirmed loss of striatal TH-immunopositive fibers in RNU−/− and RNU−/+ rat, as well as blood-brain-barrier changes associated with PD that may influence passage of immune cells into the central nervous system in RNU−/− brains. Specifically, GFAP immunopositive cells were decreased, as were astrocytic end-feet (AQP4) contacting blood vessels (laminin) in the lesioned relative to contralateral striatum. Flow cytometric analysis in 6-OHDA lesioned RNU−/+rats revealed increased CD4+ and decreased CD8+ T cells specifically within lesioned brain. These results suggest that both major T cell subpopulations are significantly and reciprocally altered following 6-OHDA-lesioning, and that global T cell deficiency exacerbates motor behavioral defects in this rat model of PD.

## Introduction

Parkinson’s disease (PD) is a progressive neurodegenerative disorder that is most common in patients over 65 years old, occurs in approximately 2 out of 1,000 people in the western hemisphere. PD is characterized by the progressive loss of midbrain dopamine neurons in the substantianigra, which causes motor dysfunction amongst other disorders. There is no cure for this disease currently, and dopamine replacement therapy has shown little efficacy. There has been growing evidence that adaptive immunity may play a role in PD progression as well as other neurodegenerative disorders including Alzheimer’s disease. In neurodegenerative diseases, the adaptive immune response may provide antigen-specific neuroprotection critical for brain repair. T cells act as the mediators of adaptive cellular immunity, allowing the body to mount increasingly potent responses to antigens of infected, transformed, or damaged self-cells with each encounter. A current debate in PD centers upon whether and how the adaptive immune response is involved in PD etiology and/or progression. Recent reports using MPTP mouse models for PD have suggested that CD4 T cells may either be protective, or promote PD-like motor behavioral symptoms. However, the exact role of T cells in aggregate (i.e., as they normally co-exist) has not been elucidated [1,2].

One possibility is that adaptive cellular immunity impairs brain tissue regeneration and health in PD patients. Neuroinflammation including reactive gliosis invariably found following brain injury/insult, is associated with PD pathogenesis and progression. The presence of reactive microglia in the substantianigra (SN) of PD patients [3], elevated levels of cytokines in striatum and dopaminergic neurons [4], and increases in T cell levels in the PD patients’ blood [5] are consistent with the view that T cells contribute to PD progression.

It has also been reported that PD patients have decreased circulating helper T cells (CD4+), while cytotoxic T cells (CD8+) are unchanged or slightly increased relative to controls [6]. Furthermore, these diminished CD4+ T cells exhibited decreased Fas expression, increased susceptibility to apoptosis [7], and increased responsiveness to nitrated-alpha-synuclein, suggesting altered responsiveness to PD-associated protein antigen [8]. Together, this suggests altered CD4+ T cell function in particular in PD patients. Regulatory CD4+ T cells, which can inhibit both CD4+ and CD8+ T cell responses, have been suggested to protect SN dopaminergic neurons from MPTP-induced cell death mice [2,8,9]. One way this could occur is by inhibiting detrimental activity of effector T cells. Indeed, CD4+ but not CD8+ T cells are required for MPTP-induced neurodegeneration in mice [2], as was FasL but not IFN-α expression, implicating T effector functions. Nevertheless, distinct models of PD may be differentially impacted by T cell subpopulations.

One way T cells could impact PD-like symptoms is by altering the transit of T cells across the blood-brain barrier (BBB) independent of their antigen reactivity, which might also serve to enhance the extravasation of activated T cells reactive against but normally sequestered from normal brain components. Consistent with this, changes in the blood-brain barrier (BBB) that could affect T cell trafficking within the central nervous system (CNS) have been observed in neurological disorders [10]. The BBB is composed of a network of astrocytic end-feet closely associated with endothelial cells that form CNS blood vessels [11–14]. Recently, several studies demonstrated alterations in the BBB utilizing animal models of PD [15–21]. However, whether and how BBB is structurally compromised in animal models of PD, and any association with T cell status, has not been elucidated.

The role of T cells in disease processes generally has been widely studied in rodents harboring a mutant Foxn1 gene, in which all T cells are reduced or absent. For example, Rowett rats, designated RNU−/−, are homozygous for a recessive mutant Foxn1 gene and exhibit a substantial reduction in all mature T cells. By contrast heterozygous RNU−/+ rats are phenotypically normal with a full complement of functional T cells. RNU−/− rats also exhibit potentially compensatory increases of systemic natural killer (NK) cells and macrophages that could complicate interpretations of immune cell involvement in studies that use them. In this context, NK cells are not typically present in large numbers in brain, but microglia constitute a unique subpopulation of resident macrophages that enter and persist in brain early in blood cell development. Indeed, microglial activation occurs in PD and its rodent models. Nevertheless, we justified studying the effects of 6-OHDA toxin administration in RNU−/− rats to determine the role of T cells in PD, because microglial activation in this PD model is transient, dependent on physical injection rather than 6-OHDA itself, and follows rather than precedes neuronal loss, all suggestive of a non-causative and toxin-independent reaction to injury [22]. We thus included analysis of time points beyond peak microglial activation in this model as well, to further ensure minimal impact by microglia.

In this study, we stereotactically administered the neurotoxin, 6-hydroxydopamine (6-OHDA) unilaterally into the striatum of outbred Rowett rats to induce PD-like lesions of the nigrostriatal dopamine system. 6-OHDA is a hydroxylated analog of dopamine, which is selectively taken up by the dopamine transporter (DAT) in dopaminergic neurons of the SN that project to the striatum. Local administration of 6-OHDA is a well-established method to induce neuropathological and neurochemical changes similar to those seen in PD patients [23]. We have chosen to use this model instead of the MPTP model because it allows comparison of the contralateral hemisphere (non-lesioned) to the lesioned hemisphere within the same animal, to determine if there are differences in T cell content or quality within lesioned and non-lesioned brain. In addition, it provides a separate PD model in which to examine the role of T cells independent from those previously examined.

We demonstrate by flow cytometry that CD4+/CD8+ T cell ratio in brains of 6-OHDA-lesioned immunocompetent rats compared to unlesioned controls is increased, which differs from findings in previous studies, but is consistent with a role for T cells in disease progression. In order to determine this role, we examined lesion severity and behavior in homozygous Rowett (RNU−/−) rats Behavioral tests confirmed that, after 6-OHDA lesioning, both RNU−/− and RNU−/+ rats favored use of the limb ipsilateral to the lesion by the cylinder test. Nevertheless, rotational tests revealed greater rotational asymmetry in 6-OHDA lesioned RNU−/− compared to RNU−/+rats, despite equivalent loss of striatal TH-immunopositive fibers induced by 6-OHDA 4- and 8-weeks post-lesion. This suggests an overall benefit of host T cells on motor function retention and/or recovery in this model. Furthermore, a histological examination of BBB structural integrity at 4-weeks post-lesion in RNU−/− rats demonstrated a marked reduction in the interaction of astrocytic end-feet on blood vessels, highlighting a possible role for BBB structural disintegration in altering immune cell traffic into lesions, and resulting in reduced CNS repair.

## Materials and Methods

### Animals and Surgical Procedures

#### Ethics Statement

All animal experiments were conducted in accordance with policies set by the Institutional Animal Care and Use Committee (IACUC) in Cedars-Sinai Medical Center (CSMC) and by NIH guidelines, CSMC IACUC protocol number 2049. Thirty-two, three month old, female rats of the HSD:RH-FOXN1 strain, Rowett nudes (RNU) were housed two to a cage with free access to rat chow and water under a 12:12 hour light-dark cycle (Harlan, Indianapolis, Indiana, USA). Phenotypically hairless rats that are homozygous (Nude) for the recessive mutant allele Foxn1, are athymic and have a deficiency in T-lymphocytes, whereas heterozygous rats (Het) retain a full complement of T-lymphocytes. All surgical procedures were performed under ketamine hydrochloride (80 mg/kg) and xylazine (10 mg/kg) anesthesia. Animals were mounted on a stereotactic apparatus (Kopf Instruments, Tujunga, CA, USA) that supports the animals’ mouth and ears for all intrastriatal injections. Eight Nude and eight Het animals each received three 2 μL injections of 3.5 μg/μl of 6-OHDA (Sigma-Aldrich Co., St. Louis, MO) dissolved in 0.05% L-ascorbic acid in 0.9% Dulbecco’s Phosphate Buffered Saline (PBS; Invitrogen Corporation, Carlsbad, California) into the Three 7-mg deposits of 6-OHDA were injected into the right lateral caudate−/+putamen (CPu). To minimize the variability of lesion caused by degradation of the toxin, the 6-OHDA was stored in the dark at −20°C and freshly prepared prior to surgery. Once made, 6-OHDA was kept on ice, protected from light and monitored for oxidation. To serve as a surgery control, eight Nude and eight Het animals received three intrastriatal 2μl injections of 0.05% ascorbic acid in 0.9% PBS to the right striatum of equal volume. A 10 μL Hamilton microsyringe fitted with a 26-gauge steel cannula was used to perform injections. All striatal injection were given at a rate of the rate of 1 μL per minute and the cannula was left in place for an additional two minutes before retraction of the needle from the animal. Relative to bregma and ventral to the dura with the tooth-bar set at 0 mm, the following lesion coordinates in the anterior-posterior (AP), medial-lateral (ML), and dorsal-ventral (DV) axes were used for each of three sites: (1) AP 1.0, ML −3.0, DV −5.0; (2) AP −0.1, ML −3.7, DV −5.0; and (3) AP −1.2, ML −4.5, DV −5.0. Dose and coordinates were selected based on prior research [24].

After striatal delivery of the 6-OHDA, the wound was closed with wound clips and animals were given subcutaneous injections of 2 mL of PBS in order to prevent dehydration and subcutaneous injections of 1 mL of carprofen (5 mg/kg) for post-operative pain management. Animals were kept under a heat lamp and a thermal blanket before and after surgery in order to maintain body temperature. When animals began to recover, animals received 0.15 cc of subcutaneously buprenorphine (0.5 mg/kg). Early and late analysis time points 4 weeks apart were chosen to determine if there was any progression in motor dysfunction and/or pathology, with late times further justified minimizing the impact of transient neuroinflammation.

### Behavioral Analysis

#### 

##### Open field test

The open field test was conducted on RNU−/− and RNU−/+ rats prior to surgery and the D-amphetamine sulfate induced rotational test in order to determine whether or not RNU−/− and RNU−/+ rats respond differently to amphetamine D-amphetamine sulfate. The open field test consists of a square arena (60 cm × 60 cm) enclosed in a Plexiglas chamber with 50 cm high walls (San Diego Instruments, San Diego, CA). Equally spaced photobeams (8 cm × 8 cm) with directly opposed photosensors were projected four inches off the floor. Behavioral activity was quantitatively and automatically measured by the number of parallel light beams that were intercepted by the animal in the field. When two adjacent light beams were intercepted (scored as single light beam breaks), the animal was deemed to have actively engaged in locomotor activity, as opposed to passive motor activity (e.g. stationary movement). Rats are assessed during a habituation (baseline) period of 30 minutes and are assessed for total ambulatory locomotion for an additional 120 minutes after intraperitoneal injection of D-amphetamine sulfate (2.5 mg/kg; Sigma-Aldrich, St. Louis, MO).

##### D-amphetamine sulfate induced rotational test

At two and six weeks post-surgery, animals that received intracranially injections of 6-OHDA or saline were monitored for rotational behavior. Beginning at 8:00 AM and after thirty minutes of habituation to the testing environment, rats were placed into a clear, Plexiglas cylinder 35 cm in diameter and 50 cm in height. Rats were placed in a wire harness with a rotary encoder that did not restrict movement (AccuScan Instruments Inc., Columbus OH, USA) and were allowed to habituate to the chamber. Animals were monitored for spontaneous rotation for fifteen minutes and then weighed and given a subcutaneous injection of D-amphetamine sulfate at a dose of 2.5 mg/kg (Sigma-RBI, St. Louis, MO). Rotational asymmetry was monitored for ninety minutes. Net rotational asymmetry score is expressed as 360° turns per min. Rotations ipsilateral to the lesioned were counted as a positive value, while rotations towards the contralateral side of the lesion were counted as a negative value. For analysis, animals treated with 6-OHDA were normalized to their saline controls.

##### Cylinder test

At four weeks and eight weeks post-surgery (2 weeks after prior testing to ensure minimal impact by drug and/or stress) forelimb akinesia and postural abnormalities that result from lesions of the basal ganglia were assessed using the cylinder test as described by Schallert et al. [25]. The cylinder test assesses rat preference in forelimb choice while rats support their body against the walls of a cylindrical enclosure as they explore the novel environment. Animals were individually placed in a transparent cylinder 25 cm in diameter and 30 cm in height. The testing room remained dark, while the Plexiglas cylinder was illuminated to aid in video recording and to stimulate the animal into exploration. The test was performed between 9:00 and 13:00 hours, and there was no prior habituation to the cylinder prior to filming. The weight bearing forelimb touches were recorded and a total of 20 touches were recorded for each animal (5−/+7 minutes). Mirrors were placed behind the cylinder at a 45° angle to allow for all forelimb touches to be visible to the observer. If the animal showed little interest in exploring the cylinder, the animal was briefly removed from the apparatus for 30 seconds, and then, replaced according to [26]. The cylinder test was filmed to allow for weight bearing paw touches (i.e. full apposition of the paws with open digits to the cylinder walls) to be validated by several observers. The values were expressed as the percentage of right, left or both forelimb touches over the total number of forelimb placements. Non-surgical animals were used as a control to ensure that no inherent forelimb preference existed.

### In-Situ Analysis

#### 

##### Animal perfusions

Immediately following the final behavioral tests, animals were deeply anesthetized with ketamine and xylazine and thoracic cavity was opened with sharp scissors, exposing the heart and ascending aorta. An 18-gauge needle secured to vacuum container collection tubing was inserted through the left ventricle. Animals were slowly perfused with cold PBS at room temperature until blood vessels were cleared. Animals were then perfused with 250 ml of ice cold, 4 % paraformaldehyde (PFA/0.1M PBS). Animals were decapitated, and brains removed.

##### Immunohistochemistry

Brains were placed in cold, 4% PFA in PBS for two hours at 4°C. Subsequently, brains were sunk in 30% sucrose and 1.25% PFA/PBS at 4°. Free-floating brain sections from the striatum and substantianigra of each treatment group were sectioned with a microtome at 40 μm thickness and rinsed in 0.1 M phosphate buffer (PB). Sections were permeabilized with 0.1% Triton-X and blocked with 5% normal goat serum (Sigma-Aldrich Co., St. Louis, MO) in 0.1 M PB for thirty minutes at room temperature. Sections were incubated overnight at 4°C with a single or triple combination of primary antibodies. The following primary antibodies were used in this study: mouse monoclonal or rabbit affinity-purified anti-TH antibody (1:1000, Chemicon International Inc., Madrid, Spain and Pel-Freez, Rogers, AK), rabbit polyclonal anti-glial fibrillary acidic protein (GFAP) antibody (1:100, Dako, Denmark), mouse monoclonal MRC OX-42 antibody against the rat equivalent of human CD11b (1:100, AbDSerotec), mouse monoclonal MRC OX-6 antibody against major histocompatibility complex (MHC) class II (1:200, Abcam Inc., Cambridge, MA), rabbit polyclonal anti-aquaporin 4 (AQP4) antibody (1:200, Chemicon International Inc., Madrid, Spain), and a chicken polyclonal antibody against laminin (1:200, Abcam Inc., Cambridge, MA). All antibodies were diluted in 0.5 mL blocking solution. Primary antibodies were detected with fluorescein (FITC)-conjugated donkey anti-chicken (Jackson Immunoresearch Laboratories, Inc., West Grove, Pennsylvania), Alexa Fluor 568 goat anti-rabbit, and Alexa Fluor 647 rabbit anti-mouse secondary antibodies (Invitrogen Corporation, Carlsbad, CA), diluted 1:200 in 0.1 M PB, for one hour at room temperature in the dark. Subsequent washes were performed in 1 mL of 0.1 M PB for five minutes. Sections were counterstained with TO-PRO-3 iodide (Invitrogen Corporation, Carlsbad, CA) in 1 mM of dimethyl sulfoxide or 4′,6-diamidino-2-phenylindole (DAPI) to label all nuclei. Sections were mounted on Superfrost plus slides (Fisher, USA) with the aid of a sable brush and coverslipped in an aqueous mounting medium (Biomeda Corporation, Foster City, CA).

##### Quantification of TH+ immunoreactive area of dopaminergic neurons in rat SN and Striatum

Total areas of TH+-immunoreactivity were determined from 3–4 coronal rat brain sections for each animal, 30 μm thick, with intervals of 150 μm, over an area covering the substantianigra region and the dorsal lateral striatum. For the striatum, free-floating sections were pretreated for 30 minutes at room temperature with 0.3% H_2_O_2_ to block endogenous peroxidase activity, washed three times with PBS, blocked with blocking solution (5% normal goat serum (Sigma-Aldrich Co., St. Louis, MO) for 1 hr at room temperature. Sections were incubated overnight at 4°C with primary antibody: affinity-purified anti-TH antibody (1:1000, Chemicon). The sections were then washed three times with PBS and incubated for 1 hour at RT with biotinylated horse anti-mouse secondary antibody (1:200) and subsequent incubation with avidin-biotin-peroxidase complex (ABC-Elite kit, Vector Laboratories). After washed three times with PBS, the reaction was visualized with 0.02% diaminobenzidine for 8 min (DAB, DAKO Liquid DAB Substrate Chromogen System). The mounted sections were dehydrated in graded ethanol solutions and embedded in DePeX mounting medium (Sigma). DAB immunoreactivity was quantified using NIH Image I software (National Institutes of Health, USA) using a standardized histogram-based threshold technique [27].

Fluorescence-specific signals within the SN were captured using confocal microscopy (Leica, USA; specified below) with the same exposure time for each image. At least 3 serial sections from >3 individual brains per group were analyzed, with inadequately stained sections (<50 contralateral TH+ cells) excluded from the analysis. Optical sections from each field of the specimen were imported into and analyzed using the NIH Image J software (National Institutes of Health, USA). Immunoreactivity was determined using a standardized histogram-based threshold technique [27]. In addition, cells that were positively labeled were individually counted using the Image J native counter using grid size 70,000, and average cell counts were compared and analyzed across all experimental groups.

##### Microscopy

Images were obtained using an upright fluorescent microscope (Axioskop2, Carl Zeiss, MicroImaging Incorporated, Thornwood, NY), a laser-scanning confocal microscope (Leica Microsystems, Wetzlar, Germany) and/or a spinning-disc confocal microscope (PerkinElmer, Waltham, MA). Image analysis was conducted with the use of Zeiss LSM 510 and Volocity 2.0 and Adobe Photoshop software.

##### Statistical analysis

Results are expressed as means −/+ standard error of the mean (SEM.) Cylinder test and rotational behavior were analyzed using T-test and post-hoc analysis. P- values less than 0.05 were considered statistically significant (*P <0.05; ***P = 0.0001 determined by one-way ANOVA or two-tail paired t-test). Prizm software was used for all statistical analysis and graph presentations.

## Results

### 6-OHDA Effects on striatal TH-positive neurons in the substantia nigra

**Figure 1:**
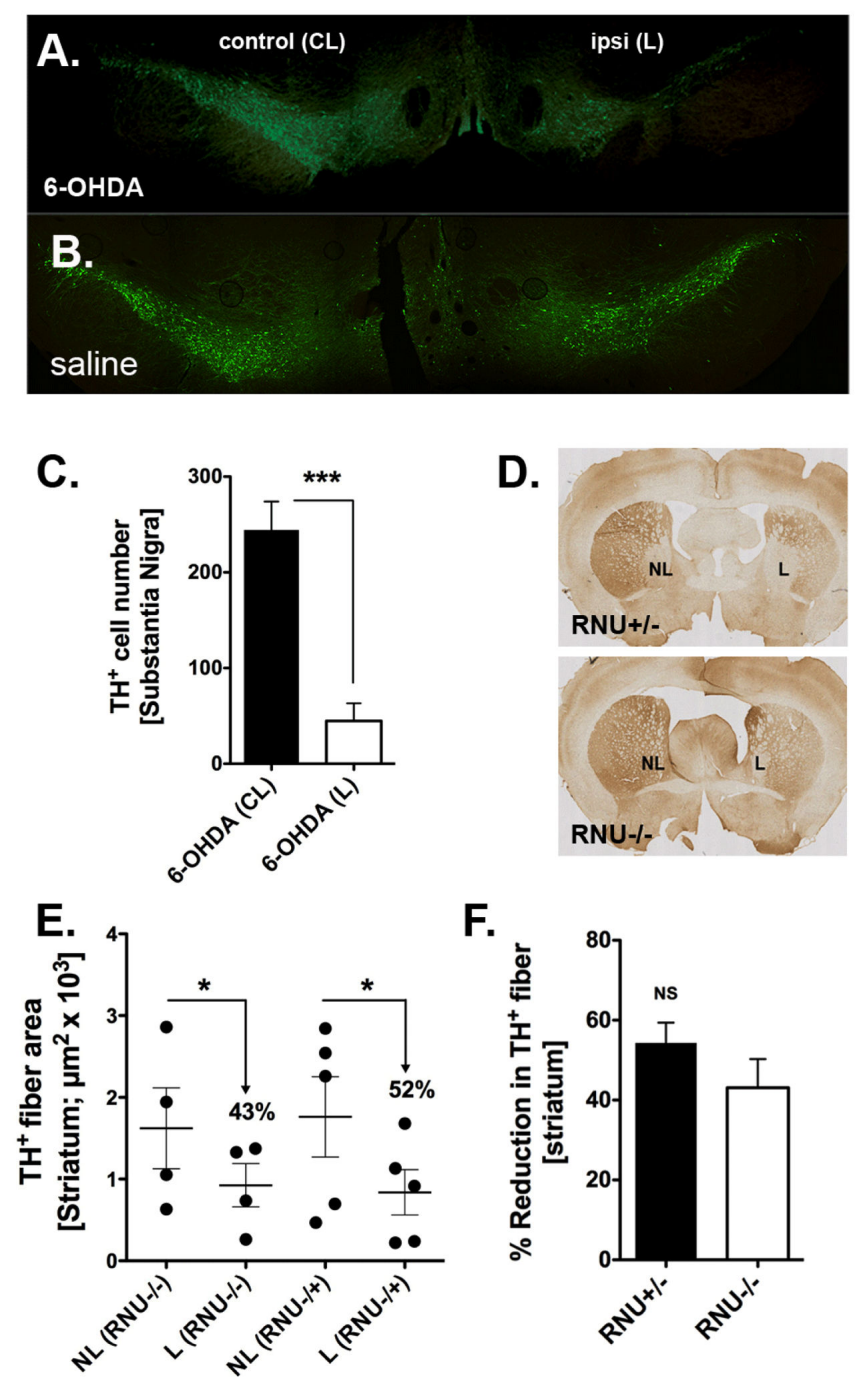
Tyrosine hydroxylase (TH) immunohistochemistry in the substantia nigra (SN) and striatum. (A) Representative immunofluorescent (IF) microphotographs demonstrating extensive loss of midbrain TH+ dopamine (DA) neuronal cell bodies (green) in the SN of RNU−/+ rats 4 weeks after unilateral 6-hydroxydopamine (6-OHDA) lesion. (B) No significant loss is observed in the SN of RNU−/+ rats following saline injection. (C) Quantitative analysis of TH-immunoreactive DA cells confirms a significant loss in cell number induced by 6-OHDA in the ipsilateral SN (ipsi lesion − L) as compared to the contralateral SN (control − CL) of RNU−/+ rats. (D) Immunohistochemical images indicate a depletion of TH-positivefiber density (brown) in the striatum of both RNU−/+ and RNU−/− rats 8 weeks after 6-OHDA injection (lesion − L,non-lesion − NL). (E) Quantitative analysis of TH+ fiber area confirms a significant reduction of ≈40–50% in striatal fiber density in the injected sites (X + SEM, Y + SEM, respectively). (F) Percent reduction of TH-fiber density after 6-OHDA injections does not differ significantly between RNU−/+ and RNU−/− rats; N = 3–4 rats per group.

To verify effective lesioning at the cellular level, we examined the extent of dopaminergic neuronal degeneration in the SN and striatum induced by 6-OHDA by TH-immunohistochemistry for both short-and long-term survival of RNU−/− and RNU−/+rats (i.e. 4 and 8 weeks post-surgery, respectively) (Figure 1A and 1B). The total number of TH-positive cells within the SN of RNU−/−and RNU−/+rats was further assessed quantitatively in 6-OHDA and saline controls (Figure 1C). Overall numbers of contralateral SN TH immunopositive cells were comparable between 6-OHDA-lesioned and saline-injected controls (244+30 and 201+16, respectively; P = 0.36), whereas a large reduction was evident in the lesioned 6-OHDA SN of both RNU−/− (not shown) and RNU−/+rats (Figure 1C). Moreover, although most TH+ cells were retained inipsilateral SN of saline controls (125+21=63% of contralateral; not shown), significantly fewer were retained in ipsilateral SN of 6-OHDA-lesioned rats (45+18 = 18% of contralateral; Figure 1C; P = 0.02 relative to saline group). No significant differences were observed in midbrain DA TH immunopositive cells by IHC between RNU−/− and RNU−/+ brains, providing a level of validation of behavioral tests that indicated comparable lesioning in both rat strains (not shown). We also sought to quantify the amount of striatal TH immunopositive fibers in both the lesioned and contralateral striatum in RNU−/− and RNU−/+ (Figure 1D–F). While we observed a significant reduction of TH immunopositive striatal fibers in the lesioned striatum as compared to the contralateral striatum we found no significant difference in the reduction of these fibers between RNU−/− and RNU−/+ rats (Figure 1E, F).

Attenuated recovery from Forelimb Asymmetry after unilateral 6-OHDA partial lesion of the nigral-striatal dopaminergic pathway in athymic rats. There are several behavioral tests to measure movement impairments in PD animal models [23,28,29]. We chose the D-amphetamine induced rotational behavior test to detect unilateral defects in DA neuronal activity, and the cylinder test, which quantifies the number of times rats use each of their forepaws to explore a cylinder. Each of these tests were performed at two time points after lesioning to examine potential progression or recovery of asymmetric motor behaviors in the presence or absence of T cells. Intrastriatal saline delivery was used to control for the injection in both RNU−/−and RNU−/+ rats and non-surgery controls were used as baseline.

**Figure 2:**
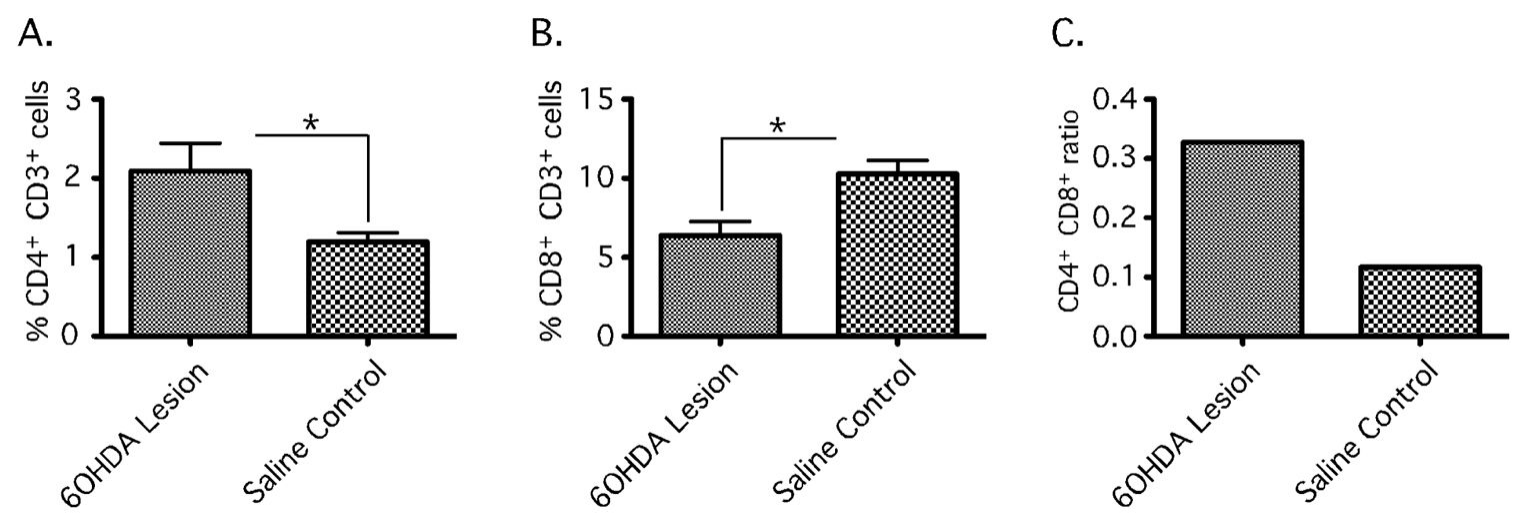
Changes in Cerebral T cell populations in the 6-OHDA lesioned RNU−/+ rat brain assessed by Flow cytometry. Striatal and nigral tissue was isolated from RNU−/+ rats 4 weeks after receiving unilateral 6-OHDA toxin or saline as control. Ipsilateral and contralateral to lesion tissue was isolated and dissociated to single cell suspensions for immunocytochemistry to identify CD3+CD4+ and CD3+CD8+ T cells. Cells were than analyzed by flow cytometry and expressed as percent of gated lymphocytes. 6-OHDA tissue showed a significant increase in CD3+CD4+ (%2.091−/+ 0.3538 vs. %1.195 −/+ 0.1160; *, P= 0.0210) immunopositive cells and decrease in CD3+CD8+ (%6.382−/+ 0.8912 vs. %10.27−/+ 0.8661; *, P=0.0034) immunopositive cells as compared to saline injected controls (A+B) and an increase in the ratio of CD4+CD8+ in 6-OHDA RNU−/+ as compared to controls (C). Error bars equal SEM (*, P<0.05).

**Figure 3:**
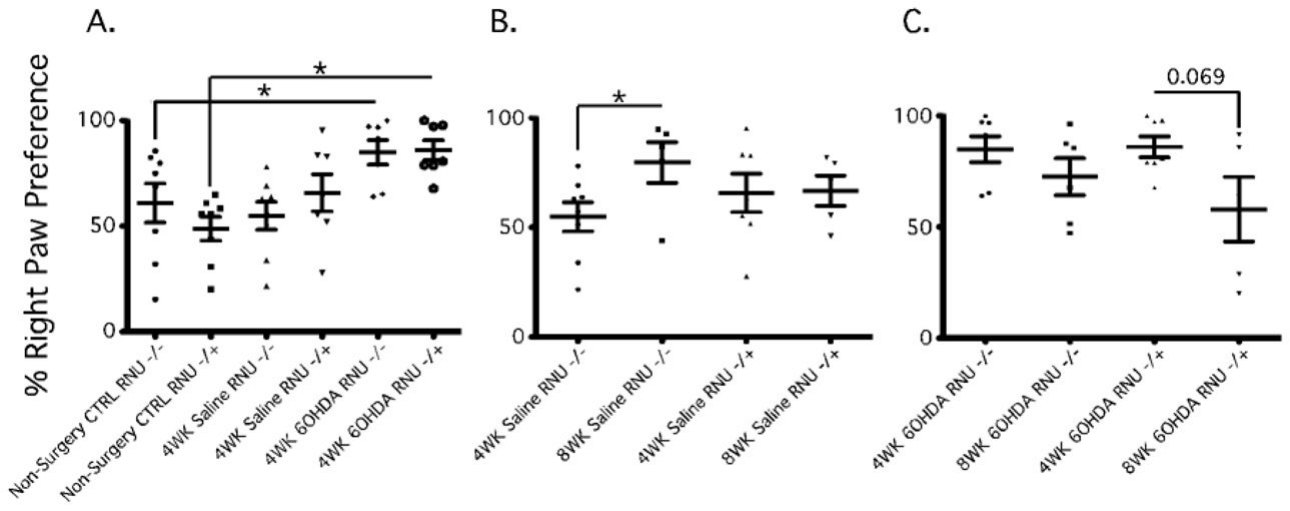
Athymic rats show attenuated asymmetric forelimb recovery by Cylinder Tests after unilateral 6-OHDA intrastriatal lesion of the nigro-striatal dopaminergic pathway. At 4 and 8 weeks post unilateral 6-OHDA lesion we used the Cylinder test to determine preferential paw. At 4 weeks post lesion RNU−/− and RNU−/+ showed a significant preferential right paw use as compared to non-surgery controls RNU−/−(%60.87 −/+ 9.249 vs. %84.91 −/+ 5.814; *, P = 0.026); RNU−/+ (48.69 −/+ 5.618 vs. 85.94 −/+ 4.685; *, P = 0.0002) (A). At 8 weeks post saline RNU−/− showed significant right paw preference as compared to 4 wks post saline (%54.81 −/+ 6.614 vs. 79.60 −/+ 9.261; *, P = 0.0236) (B). At 8 weeks post 6OHDA lesion RNU −/+ showed a trend towards recovery from right paw preference (%57.94 −/+ 14.49 vs. 85.94 −/+ 4.685; P = 0.069), while RNU−/− shows no significant change in right paw use (%72.54 −/+ 8.286 vs. 84.91 −/+ 5.814; P = 0.2529). Error bars equal SEM (*, P<0.05).

The cylinder test was performed at four and eight weeks post 6-OHDA lesioning. At four weeks the cylinder test revealed no significant differences in forelimb use between RNU−/− and RNU−/+ rat saline control groups and non-surgery controls (Figure 2). 6-OHDA lesioned RNU−/− and RNU−/+ rats, however, both exhibited a preference to use the forelimb ipsilateral to the lesion (i.e. the right forelimb) as compared to saline control groups (Figure 3), with no significant difference in right paw use observed (Figure 3). At eight weeks post-surgery, both RNU−/− and RNU−/+ rats with 6-OHDA lesions continued to exhibit a significant preference to use the right forelimb (Figure 3), but at this time point RNU−/+ rats showed a trend towards reduced right paw use after lesioning, whereas RNU−/− rats did not. These results suggest that 6-OHDA lesioned RNU−/− and RNU−/+ rats equally preferred to use the forelimb ipsilateral to the lesion, but that T cell deficiency may reduce intrinsic recovery from asymmetric forelimb use. Moreover, saline injected RNU−/− controls showed a significant increase in right paw use after 8 weeks (Figure 3), suggesting that T cell deficiency may exacerbate asymmetric forelimb use caused by physical damage from saline injection into the striatal parenchyma over time.

### Delayed recovery of rotational motor behavior in athymic rats

**Figure 4:**
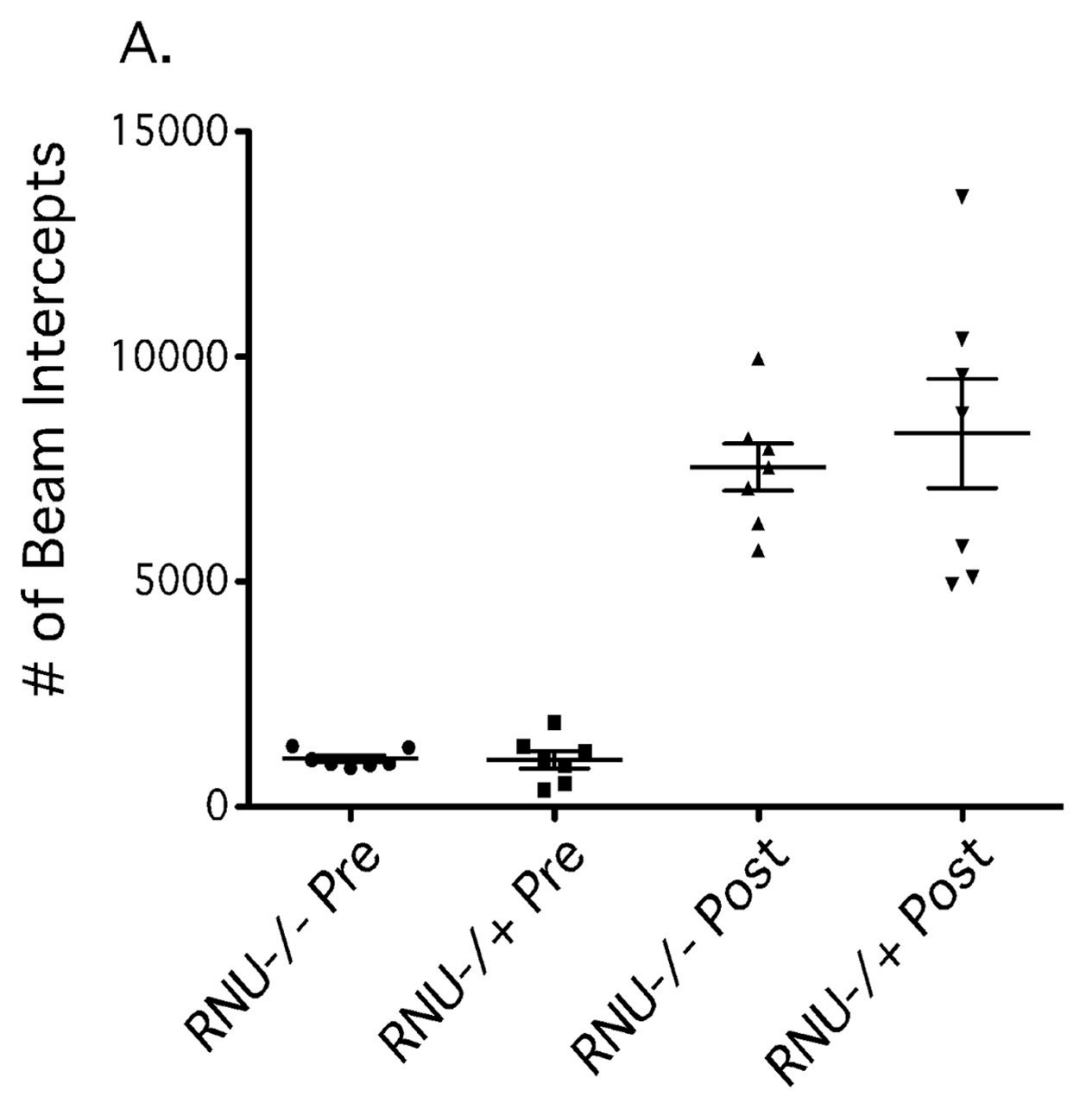
Open Field test of Total Ambulatory and Central locomotor activity in RNU−/− and RNU−/+ after D-amphetamine sulfate administration. In order to assess any potential differences in the metabolism of the drug, D-amphetamine sulfate, and subsequent locomotor activity between RNU−/− and RNU−/+ rats, an Open Field test was conducted prior to surgery (i.e. prior to saline or 6-OHDA injection). We found no significant differences in overall ambulatory (A) during a 120-minute time interval. Error bars equal SEM (*, p=0.05).

To further determine whether T cells are involved in lesion-induced pathology after 6-OHDA delivery, we aimed to quantify amphetamine-induced rotational testing in T cell-deficient RNU−/−and immune competent control RNU−/+ rats. As a prelude to this we sought to assess whether D-amphetamine sulfate is metabolized differently and leads to differences in locomotor activity between RNU−/− and RNU−/+ rats. We therefore conducted an Open Field test of locomotor activity prior to lesion surgery (e.g. 6-OHDA injection) to analyze locomotor activity. We found no differences in baseline ambulatory activity between RNU−/− and RNU−/+ rats as measured by light beam interceptions during a 30-minute interval (Figure 4), and no differences after i.p. delivery of D-amphetamine sulfate over a 90-minute time interval (Figure 4). This indicates that D-amphetamine sulfate elicits similar locomotor effects in RNU−/− and RNU−/+ rats overall, and suggests similar metabolism of the drug in these two strains. Thus, any lesion-induced differences in D-amphetamine-induced rotational behavior can be ascribed to specific strain characteristics independent of drug metabolism (i.e., T cell-deficiency).

**Figure 5:**
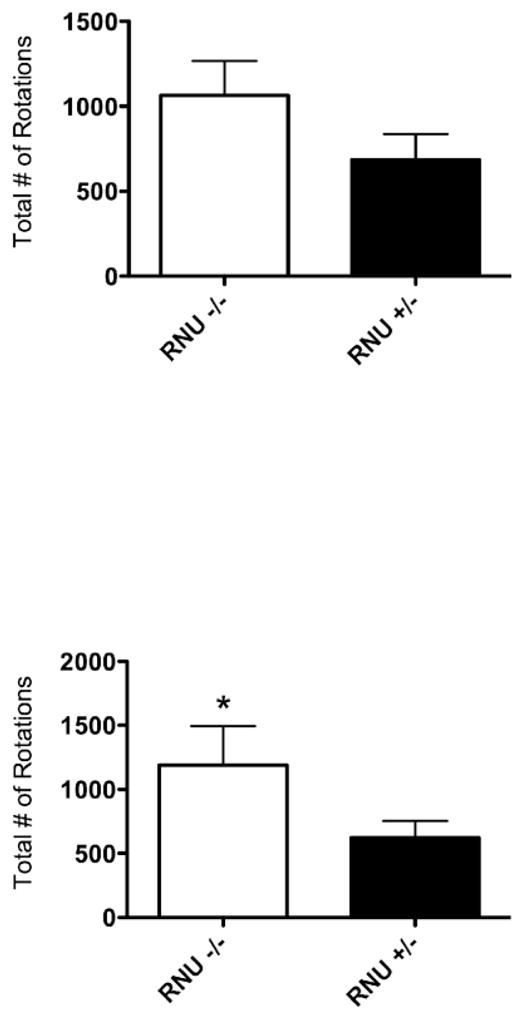
Athymic rats exhibit greater D-Amphetamine-induced rotational behavior following unilateral 6-OHDA striatal lesion of the nigro-striatal pathway. Two weeks afterunilateral 6-OHDA lesioned (right) RNU−/− rats show a trend towards increased rotational behavioral ipsilateral to the lesion (right) after d-amphetamine sulphate administration as compared to RNU−/+ rats (1064 −/+ 203.4, N=5 vs. 685.4 −/+ 151.7, N=8; *, P = 0.071) (A). At six weeks post-lesion there was a significant increase in RNU−/− rotational behavior as compared to RNU−/+rats (1188 −/+ 306.5, N=5 vs. 622.8 −/+ 129, N=8; *, P = 0.037) (B). Error bars equal SEM (*, p=0.05).

D-amphetamine sulfate-induced rotation was quantified at 2 and 6 weeks post-surgery. At 2 weeks, 6-OHDA lesioned RNU−/− rats exhibited a trend toward more counterclockwise rotations relative to RNU−/+rats (Figure 5). At 6 weeks, RNU−/− rats maintained this level of directional rotation, whereas counter clockwise rotations were diminished in RNU−/+ rats (Figure 5). This resulted in a significant increase in the number of rotations by RNU−/− relative to RNU−/+ rats only at 6 weeks post lesioning. These data suggest that T cell deficiency counteracts intrinsic recovery from rotational behavioral deficits in 6-OHDA lesioned rats. Thus, T cells may be involved in recovery from such deficitsover time.

#### Unilateral 6-OHDA striatal partial lesion promotes T cell infiltration in brain

**Figure 6:**
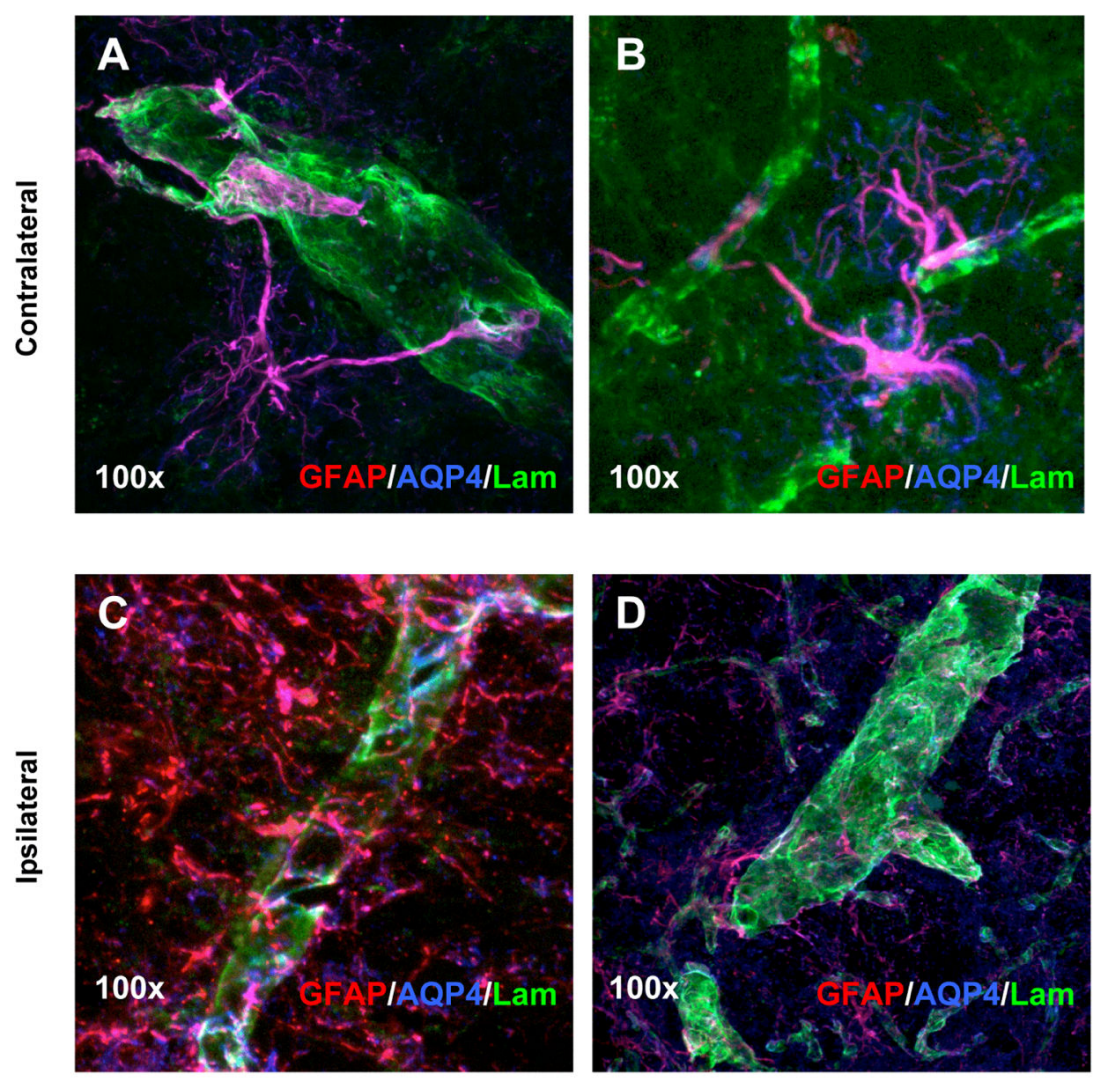
Immunohistochemistry and blood-brain barrier analysis in 6-OHDA lesioned RNU−/− rats. Laser-scanning confocal microscopy images of triple immunostained tissues using antibodies against GFAP (red), AQP4 (blue) and laminin (green) indicate BBB changes between the contralateral (A and B) and 6-OHDA lesioned brain (C and D) in RNU−/− rats. Localized morphological and protein expression changes suggests differences in the interaction between astrocytes and blood vessels 4 weeks post 6-OHDA lesion between the contralateral and ipsilateral side in RNU−/−. Magnification at 100× indicated direct association of astrocytic end-feet on blood vessels (A and B). However, AQP4 immunoreactivity was diminished and not localized to blood vessels (laminin/green) in the lesioned tissue (C and D).

It’s possible that systemic T cells could non-specifically or indirectly impact motor symptoms in the 6-OHDA model. Moreover, it was unclear from RNU rat studies how T cell subsets might be distinctly involved. In order to corroborate direct T cell involvement in 6-OHDA-mediated pathology, we determined CD4+ and CD8+ T cell levels within ipsilateral and contralateral striatal and SN tissue from brains of immunocompetent RNU−/+ 6-OHDA lesioned rats by flow cytometry. We observed a significant increase in CD3+CD4+ cells, and a significant decrease in CD3+CD8+ cells, in 6-OHDA-lesioned relative to saline-treated control brain, resulting in an increased CD4+ to CD8+ T cell ratio in the former (Figure 6). This finding demonstrates a reciprocal effect of 6-OHDA lesioning on each of the two major T cell subpopulations within the brain parenchyma, revealing the possibility that T cells may directly impact brain function in this model.

### Effects on the blood-brain barrier

Our findings of increased CD4+/CD8+ T cell ratio within brain and exacerbation of motor symptoms in T cell deficient rats suggest that T cell subsets may impact PD in more complex manners than previously thought. In this context, altered T cell ratios may reflect differential subset activation and/or entry into brain. We therefore asked whether routes of CNS access were altered by examining blood-brain-barrier (BBB) integrity, as the main route of CNS access by non-activated lymphocytes, using immunohistochemical staining of proteins on known BBB constituents in 6-OHDA-lesioned and saline-treated RNU−/+ rats. Antibodies against Glial Fibrillary Acidic protein (GFAP) expressed by astrocytes, Aquaporin-4 (AQP4) expressed in the end-feet of astrocytes that participate in the BBB, and laminin expressed by vascular endothelial cells were utilized to determine if there were any differences in expression within 6-OHDA-lesioned relative to contralateral non-lesioned brain. Laser-scanning confocal microscopy images of triple antibody immunopositive cells showed differences in protein distribution and interaction between astrocytes and blood vessels 4 weeks after 6-OHDA lesioning RNU−/+. Confocal microscopy showed direct association of astrocyticendfeet and blood vessels contralateral to the lesion. However, no direct association between astrocytic end-feet and blood vessels was observed in the lesioned tissue, as there was minimal AQP4 immunopositive labeling. This suggests that the BBB is grossly disrupted in lesioned rats, which may contribute to locally altered entry of T cell subsets.

## Discussion

Our goal was to examine whether T cell deficiency affects PD progression, and changes the BBB structure that could conceivably affect their extravasation. We observed that T cell subpopulations were modulated in a complex manner after 6-OHDA administration, with CD4+ T cells increasing and CD8+ T cells decreasing. From this observation, it was difficult to discern whether any single T cell subpopulation was particularly affected by 6-OHDA, or particularly influenced its neurotoxicity. We thus tested whether T cells in general benefitted or exacerbated 6-OHDA-induced pathology using three sets of assays to monitor lesion progression at the behavioral level: the open field, cylinder, and D-amphetamine sulfate-induced rotational asymmetry tests.

In order to assess forelimb preference in our 6-OHDA rat model for Parkinson’s disease (PD), the cylinder test was conducted as described previously[26]. In our model, DA neurons that secrete dopamine are lost following injection of 6-OHDA into the right striatum, resulting in asymmetric impairment of left forelimb use (i.e., ipsilateral limb use preference). As a result, the left forelimb is not used independently for support or stepping movements on the walls of the cylinder. Accordingly, non-surgery control rats did not exhibit a forelimb preference, whereas both RNU−/− and RNU−/+ rats injected with 6-OHDA favored right forelimb use when initiating weight-shifting movements during cylinder or environment exploration. These results suggest that 6-OHDA lesions were functionally induced and successfully created a hemi-parkinsonian model. Notably, however, no statistical significance was observed between T cell-deficient RNU−/−and immunocompetent RNU−/+ rats, suggesting that T cells did not alter overall performance in this test.

Drug-induced rotation has constituted the standard measure of behavioral outcome in unilateral 6-OHDA lesion studies and is considered the most sensitive assessment of lesion severity [26]. In the D-amphetamine sulfate induced rotational test, animals rotate toward the side of the brain with less nigro-striatal DA neuronal activity.

Open field test results indicated that both RNU−/− and RNU−/+ rats responded with equal locomotor activity in response to D-amphetamine sulfate. Therefore, to correct for potential differences in more subtle locomotor behavior, subsequent behaviors with D-amphetamine were normalized to their respective strain control groups before comparing between strains. In this context, both RNU−/− and RNU−/+ rats with a 6-OHDA lesion rotated ipsilateral to the lesion and exhibited greater rotational asymmetry under the influence of D-amphetamine relative to saline controls, indicating an effective lesion to the right side. RNU−/− rats injected with 6-OHDA at two and six weeks post-lesion exhibited greater rotational asymmetry than RNU−/+ rats, however, suggesting persistent nigro-striatal pathway and subsequent motor asymmetry deficits in RNU−/−. In contrast, RNU−/+ rats showed some decreased rotational asymmetry at 6 weeks relative to 2 weeks post-lesion, suggesting that T cell deficiency contributes to a more severe behavioral lesion after 6-OHDA administration. Hence, T cells may be involved in recovery from the behavioral effects of such administration. In this context, a recent study suggested that CD4+ T cells promote neurodegeneration in the MPTP model of PD in mice [2,8,30–32]. However, the specific impact of the any T cell subset has not been elucidated in the 6-OHDA model, which could be distinctly susceptible to immune influence.

Parkinson’s patients exhibit decreased peripheral CD4 and elevated γ/δ T cells in their peripheral blood and cerebrospinal fluid (CSF) [7,33]. In addition, significant increases in innate immune complement proteins and cytokines (e.g., IL-1, IL-2, IL-6, and TNF) within the substantianigra and CSF of PD patients has been observed [31,34]. Despite such circumstantial evidence, the overall contribution of adaptive immune processes and cells to neurodegeneration in PD is just beginning to be elucidated. In the MPTP mouse model for PD, CD4+ T cells appear to promote neurodegeneration [2]. Whereas CD4+CD25+ T regulatory cells, which inhibit effector CD4+ T cell responses, may also protect DA neurons [31], both pointing to a detrimental role for effector CD4+ T cells in this model of neurodegeneration. Notably, neither CD8+ T cells nor pro-inflammatory cytokine (IFN-α) appears to contribute to the disease phenotype in the MPTP model [2].

In our study, T cell deficiency promoted more severe behavioral deficits after 6-OHDA administration, reflecting an opposite effect than seen in the MPTP model in Rag-1-deficient or Tcrb-deficient mice [2]. This apparent discrepancy may reflect distinct sensitivity of our model to beneficial T cell functions, but could also reflect analytical differences. For example, we performed behavioral assays starting at 2–8 weeks post-lesion, which is significantly later than previous reports examining the role of T cells on behavior in rodent PD models [1,2]. In this way, we were able to assess the involvement of T cells at later disease stages, at times when microglial activation (part of the innate immune response) has begun to decline. This is important because the physical damage caused by injecting the toxin into the striatum may elicit such innate immune responses that nevertheless diminish after a few weeks. These early innate responses may distinctly effect behavior, and may be in kind distinctly impacted by T cells.

It should be noted that IHC analysis confirmed the progressive loss of dopaminergic (TH immunopositive) neurons in the SN in both RNU−/− and RNU−/+ rats after lesioning. Nevertheless, there was no detectable difference in the extent of neuronal loss in these two strains. Further studies will focus on more accurately quantifying and pinpointing neuronal loss in these strains, to further determine how and where T cells exert their influence on PD pathophysiology,

T cells may either directly affect DA neurons through cytotoxicity, or indirectly affect them through cytokine production and/or interaction with microglia or other innate immune cells [35]. It should be noted that in this report we have utilized a neurotoxin that acutely kills DA neurons as a model for PD. However, Parkinson’s disease in humans is believed to be a chronic condition developing over years. These differences add additional complexity to discerning the precise role of T cells in human disease and neurodegeneration. In addition, previous studies have demonstrated that humoral responses are present in both human and animal models for PD [36–38]. In several cases antibodies directed towards neurotransmitters and DA neuronal intracellular components, including alpha-synuclein have direct effects on DA neuronal survival [39]. Therefore, we have only begun to systematically elucidate the potential role of adaptive immunity and innate immunity and it will become essential to design future studies that interrogate these various components of the immune system and their potential interactions in order to fully understand immune involvement PD.

Although further study is necessary to determine how T cells facilitate their apparent benefit in our 6-OHDA model of PD, possibilities include the secretion of cytokines that act as neurotrophic factors to promote dopaminergic neuron maturation and survival [4]. One candidate cytokine in this respect is human IL-10, which has been shown to protect DA neurons in vitro from oxidative stress [40].

It is similarly unclear how 6-OHDA toxicity elicits differential effects on T cell subpopulations within lesioned CNS. It has been proposed that misfolded α-synuclein can be released from damaged DA presynaptic terminals and cell bodies and activate nearby microglia [41,42]. Activated M1 microglia can influence T cells, which reciprocally maintain the M1 microglial phenotype and thereby support the release of reactive NO and O_2_ to further injure DA neurons. Thus, the interplay between neurotoxicity, and innate and adaptive immune cells may be complex, further highlighting the importance of validating findings from rodent models in clinical patients.

One aspect of PD pathophysiology that could dramatically influence the presence and influence of T cells generally, and discrete T cell subpopulations specifically, is functional compromise of the blood-brain barrier (BBB)[43]. In MPTP mouse models of PD, BBB function is altered transiently, with its integrity recovered within a week [2]. Nevertheless, whether the BBB is structurally compromised in PD models has not previously been addressed. For these reasons, we sought to determine the extent of gliogenesis and determine whether structural BBB alterations associated with leakage were present and persisted in our model as a potentially major route for T cell modulation. Immunohistochemical analysis demonstrated disrupted astrocyticendfeet contact points on blood vessels within 6-OHDA-lesioned but not saline-treated striatum 4 weeks post-lesioning in RNU−/+ rats that was maintained at least 4 more weeks (not shown). These data suggest that our invasive 6-OHDA model, unlike the non-invasive MPTP model, is associated with long-term BBB disruption, and in that respect may be more representative of human PD. Although further study is warranted to determine the relevance of these changes, structural disintegration of the BBB could conceivably allow peripheral T cells to enter or leave brain tissue and thereby elicit complex effects on neurodegeneration.

General T cell deficiency, which we show exacerbates behavioral pathology in our PD model, may influence neuronal injury and tissue damage directly through reacting to self antigens in the brain, or indirectly by promoting inflammation. Inflammation can be attributed to many factors, some of which include the innate immune response and the prolonged production of pro-inflammatory cytokines, which may further compromise the health of PD patients [44, 45]. Microglia, which can produce or promote production of pro-inflammatory cytokines, tend to be present in a quiescent state in a healthy CNS, but during a diseased state (e.g. when bacteria or viruses invade) or CNS injury, these microglia become “activated,” and undergo cellular and morphological changes [46, 47]. Activated microglia have been shown to engage both innate and adaptive immune responses, enhancing phagocytosis and pro-inflammatory cytokine production as well as activating T-cells through cell surface antigens, respectively, depending upon the environment [46, 47]. Uncontrolled activation of microglia has been shown to contribute to neurotoxicity by the release of inflammatory cytokines, nitric oxide, and superoxide [48] Future research will determine how discrete innate and adaptive immune cell subpopulations interact to affect neurodegeneration in our model, possibly in conjunction with BBB alterations.
